# Pathogenicity and Aggressiveness of Corticioid Basidiomycetes Associated with Stem and Branch Rot of Avocado

**DOI:** 10.3390/pathogens15030244

**Published:** 2026-02-25

**Authors:** José Julio Rodríguez-Aguilar, Juan Mendoza-Churape, Erwin Saúl Navarrete-Saldaña, Yurixhi Atenea Raya-Montaño, Margarita Vargas-Sandoval

**Affiliations:** 1 Facultad de Agrobiología “Presidente Juárez”, Universidad Michoacana de San Nicolás de Hidalgo, Uruapan 60170, Michoacán, Mexico; 1824000b@umich.mx (J.J.R.-A.); juan.churape@umich.mx (J.M.-C.); yurixhi@umich.mx (Y.A.R.-M.); 2 Colegio de Postgraduados, Campus Montecillo, Texcoco 56264, Estado de México, Mexico; navarrete.erwin@colpos.mx; 3 Facultad de Biología, Universidad Michoacana de San Nicolás de Hidalgo, Morelia 58030, Michoacán, Mexico

**Keywords:** woody tissue diseases, ligninolytic fungi, white rot decay, *Dentocorticium portoricense*, disease progression

## Abstract

Woody tissue diseases of avocado (*Persea americana* Mill. var. *Hass*) pose a major phytosanitary threat due to their chronic progression, late symptom expression, and severe impact on tree stability and productivity. Although white rot has traditionally been attributed to saprobic basidiomycetes, increasing evidence suggests corticioid fungi may act as facultative pathogens in agricultural systems. This study examined corticioid basidiomycetes associated with white rot in stems and branches of avocado in Michoacán, Mexico. Field surveys revealed consistent symptoms of structural weakening, branch dieback, and wood decay. Fungal isolates obtained from symptomatic tissues and sporomes were characterized morphologically and identified through ITS-based phylogenetic analyses. Representative isolates of *Grammothele* spp. and *Dentocorticium portoricense* were evaluated in pathogenicity assays under controlled conditions. All isolates reproduced field symptoms, confirming pathogenicity, though aggressiveness varied. *D. portoricense* exhibited the highest incidence, severity, and AUDPC values, indicating greater virulence, while *Grammothele* isolates showed slower, moderate progression. Phylogenetic analyses provided robust support for *D. portoricense*, whereas *Grammothele* was resolved at genus level. Integration of field, pathogenicity, and molecular data demonstrates corticioid fungi are not merely secondary saprotrophs but relevant pathogens in avocado white rot. These findings highlight the need to include corticioid fungi in diagnostic, monitoring, and management strategies for trunk and branch diseases.

## 1. Introduction

Stem and branch diseases represent one of the major phytosanitary constraints in avocado (*Persea americana*) production systems, as they compromise vascular integrity, structural stability, and long-term tree productivity [1,2]. Symptoms affecting branches and trunks commonly include cankers, tissue necrosis, wood decay, and progressive wilting, leading to the loss of productive limbs and, in severe cases, the death of the entire tree [3,4]. Traditionally, these symptoms have been mainly associated with a limited group of phytopathogenic fungi, particularly members of the family Botryosphaeriaceae, as well as species of *Colletotrichum*, *Fusarium*, and other ascomycetes commonly linked to trunk and branch diseases in avocado [5,6].

However, in recent years, members of Basidiomycota have been increasingly recognized as playing a relevant role in stem and branch diseases, contrary to previous assumptions [7,8]. Within this phylum, corticioid fungi constitute a morphologically and ecologically diverse group characterized by resupinate basidiomata and a close association with woody substrates. Nevertheless, they have been scarcely studied from a phytopathological perspective [9]. For decades, these organisms were regarded exclusively as saprophytes, restricted to the decomposition of dead wood and organic residues. This historical perception led to their systematic exclusion from phytopathological studies and from routine disease diagnostics in agricultural crops [10,11].

Nonetheless, several studies have demonstrated that certain corticioid fungi are capable of colonizing living woody tissues, causing structural degradation, vascular dysfunction, and tissue necrosis in a range of host plants [7,12]. These findings challenge the traditional view of corticioid fungi as strictly saprophytic organisms and suggest a broader ecological amplitude that includes facultative pathogenic behavior. Despite this evidence, in agricultural systems these fungi are still commonly interpreted as secondary or opportunistic colonizers, particularly when isolated from infected or decaying tissues [13].

As a consequence, the pathogenic potential, epidemiological relevance, and contribution of corticioid fungi to the severity of stem and branch diseases in woody crops such as avocado remain poorly documented. This lack of recognition limits diagnostic accuracy and hinders the development of effective phytosanitary management strategies, particularly in complex pathosystems where multiple fungal agents coexist [4,14].

Recent observations in avocado orchards exhibiting symptoms of stem and branch rot have revealed the recurrent presence of corticioid fungi associated with colonized and degraded tissues. Preliminary evaluations suggest that some of these fungi are capable of actively colonizing healthy tissues and inducing disease symptoms under favorable conditions; however, systematic studies assessing their pathogenicity and aggressiveness, supported by quantitative analyses of disease progression, are still limited [15,16].

In this context, the objective of the present study was to characterize corticioid fungi associated with stem and branch rot symptoms in avocado and to evaluate their pathogenicity under controlled conditions. Disease development was quantified using incidence, severity, and the area under the disease progress curve (AUDPC), allowing a comparative assessment of isolate aggressiveness over time. The results obtained contribute to a better understanding of the role of corticioid fungi in stem and branch diseases of avocado and highlight their relevance as emerging or previously underestimated pathogens in woody crop systems.

## 2. Materials and Methods

### 2.1. Study Area and Field Sampling

Sampling was conducted in commercial avocado orchards located within the avocado-producing belt of Michoacán State, Mexico (Table 1). Trees exhibiting visible symptoms of stem and branch diseases were selected, including white rot, cortical necrosis, cankers, branch dieback, and the presence of corticioid-type basidiomata. Sampling was directed toward symptomatic trees, following methodologies commonly employed in studies of woody tissue diseases and lignicolous fungi [17].

Six representative municipalities within the region were surveyed (Table 1), with two to four orchards visited per municipality. In each orchard, three to six affected trees were selected, resulting in a total of 72 sampled trees and approximately 95 woody tissue samples and associated basidiomata.

### 2.2. Isolation and Establishment of Pure Cultures

Woody tissue samples were surface-disinfested by immersion in 70% ethanol for 30 s, followed by immersion in 1% sodium hypochlorite for 1 min, and rinsed three times with sterile distilled water. Subsequently, 5 mm tissue fragments were plated onto potato dextrose agar (PDA) and incubated at 25 ± 2 °C for 36–48 h. Isolates exhibiting morphological characteristics consistent with corticioid fungi were subcultured through hyphal tip transfer until pure cultures were obtained.

### 2.3. DNA Extraction and Identification Through Phylogenetic Reconstruction

Genomic DNA was extracted from mycelium grown for 10 days using the MLO protocol described by Osuji et al. [18], combined with 2% CTAB. DNA quality and concentration were assessed spectrophotometrically using a NanoDrop™ instrument (Thermo Scientific, Waltham, MA, USA). Amplification of the ribosomal DNA internal transcribed spacer (ITS) region was performed by PCR using the primers ITS5 and ITS4 [19]. Amplification products were verified by electrophoresis on 1.5% agarose gels. A PCR cleanup step was perfomed to remove remaining primers using ExoSAP and commercially sequenced. The resulting sequences were compared with those available in GenBank using the BLAST tool (BLAST+ 2.17.0) of the National Center for Biotechnology Information (NCBI).

For phylogenetic analyses, a reference sequence dataset was constructed using sequences retrieved from NCBI, with taxonomic nomenclature verified through the MycoBank online platform. Sequence alignments were performed using MAFFT v7 and manually edited. The best-fitting evolutionary model was selected using jModelTest v2.1.10. Phylogenetic analyses were conducted using Bayesian inference in MrBayes v3.2 and maximum likelihood in RAxMLGUI. Phylogenetic trees were visualized and edited using MEGA v7 and FigTree v1.4.5.

### 2.4. Strain Preservation and Inoculum Preparation

Fungal isolates were preserved on sterilized sorghum grains autoclaved at 121 °C (15 psi) for 2 h and stored at 4 °C until use. Inoculum was prepared by inoculating actively growing mycelial plugs (7-day-old cultures) onto hydrated sorghum grains, which were then incubated at 28 °C until complete colonization.

### 2.5. Pathogenicity Tests

Experimental avocado plants were grown under greenhouse conditions. All plants originated from seeds collected from a single mother tree and were subsequently grafted with scions obtained from a single Hass donor tree, in order to minimize genetic variability. At the time of inoculation, plants were 11 months old, showed uniform growth, and did not exhibit visible symptoms of trunk or branch diseases.

Artificial inoculations were conducted on both trunks and branches. Prior to inoculation, the bark surface was disinfected with 70% ethanol. Circular wounds approximately 5 mm in diameter and ~2 mm deep was made using a sterile cork borer, carefully removing the bark to expose the xylem tissue without causing extensive mechanical damage.

Inoculation sites on the trunk were established on the main stem, while branch inoculations were performed on secondary branches of comparable diameter. All wounds were made on healthy tissue, avoiding natural cracks, lenticels, or pre-existing injuries.

Agar plugs (5 mm diameter) taken from the actively growing margin of 7-day-old fungal cultures were placed mycelium-side down onto the exposed xylem. The inoculation sites were covered with sterile moist cotton and sealed with Parafilm^®^ to prevent desiccation and external contamination. Control plants were treated identically, using sterile PDA plugs.

Inoculated plants were maintained under greenhouse conditions and monitored periodically for symptom development. At the end of the evaluation period, tissues from inoculated sites were collected, and the fungi were re-isolated to fulfill Koch’s postulates.

Disease development was monitored every 48 h for 125 days after inoculation. At each evaluation, lesion size was quantified by measuring lesion length (L, cm) along the main longitudinal axis of the stem or branch and maximum lesion width (W, cm) perpendicular to L at the widest point, using a ruler or digital caliper.

Lesion area (A, cm^2^) was estimated using an elliptical approximation according to the formula:

A = π/4 × (L × W),

where L corresponds to lesion length and W to maximum lesion width. This approach was used to provide a consistent and reproducible estimation of lesion expansion over time.

In addition to lesion measurements, fungal signs were recorded as qualitative observations throughout the evaluation period. The presence of visible mycelial growth was assessed both superficially on the bark and internally, after careful removal of the bark to expose the underlying xylem tissue. Internal colonization was identified by the presence of white to cream mycelial growth within discolored wood. These observations were documented photographically and used as supporting evidence of active fungal colonization, but not as primary criteria for severity scoring.

### 2.6. Disease Assessment and Statistical Analysis

Disease severity was assessed using an ordinal scale from 0 to 5, primarily based on lesion length and associated tissue alterations: 0 = no visible symptoms; 1 = very slight lesion (L ≤ 1.0 cm) limited to the wound margin, without evident xylem discoloration; 2 = mild lesion (L > 1.0–2.5 cm) with initial xylem discoloration; 3 = moderate lesion (L > 2.5–4.5 cm) with evident xylem browning around the inoculation point; 4 = severe lesion (L > 4.5–6.5 cm) with pronounced xylem discoloration extending beyond the inoculation site and incipient tissue softening; 5 = very severe symptoms (L > 6.5–8.0 cm), characterized by extensive necrosis, marked xylem discoloration, and clear degradation of woody tissue, frequently accompanied by visible superficial and/or internal mycelial growth.

Disease progress over time was summarized by calculating the Area Under the Disease Progress Curve (AUDPC) for lesion length (and lesion area when applicable) using the trapezoidal integration method.

Aggressiveness data were analyzed using one-way analysis of variance (ANOVA), followed by Tukey’s test (*p* ≤ 0.05). Statistical analyses were performed using the R software (R version 4.5.2) environment, employing the agricolae package.

## 3. Results

### 3.1. Symptoms Observed in the Field

In the evaluated avocado orchards, trees exhibiting consistent symptoms of white rot affecting woody tissues were observed during the annual cycles from 2022 to 2025, primarily on stems and branches. Symptoms included structural weakening, mechanical fractures, bark fissures, and exposure of degraded wood. In both transverse and longitudinal sections, affected tissues exhibited a whitish to creamy-white discoloration, a marked loss of hardness, a fibrous to spongy texture. Structural alteration of the xylem, with diffuse boundaries between healthy and diseased tissues. No signs associated with brown rot, such as generalized darkening or cubical cracking of the wood, were observed.

**Figure 1:**
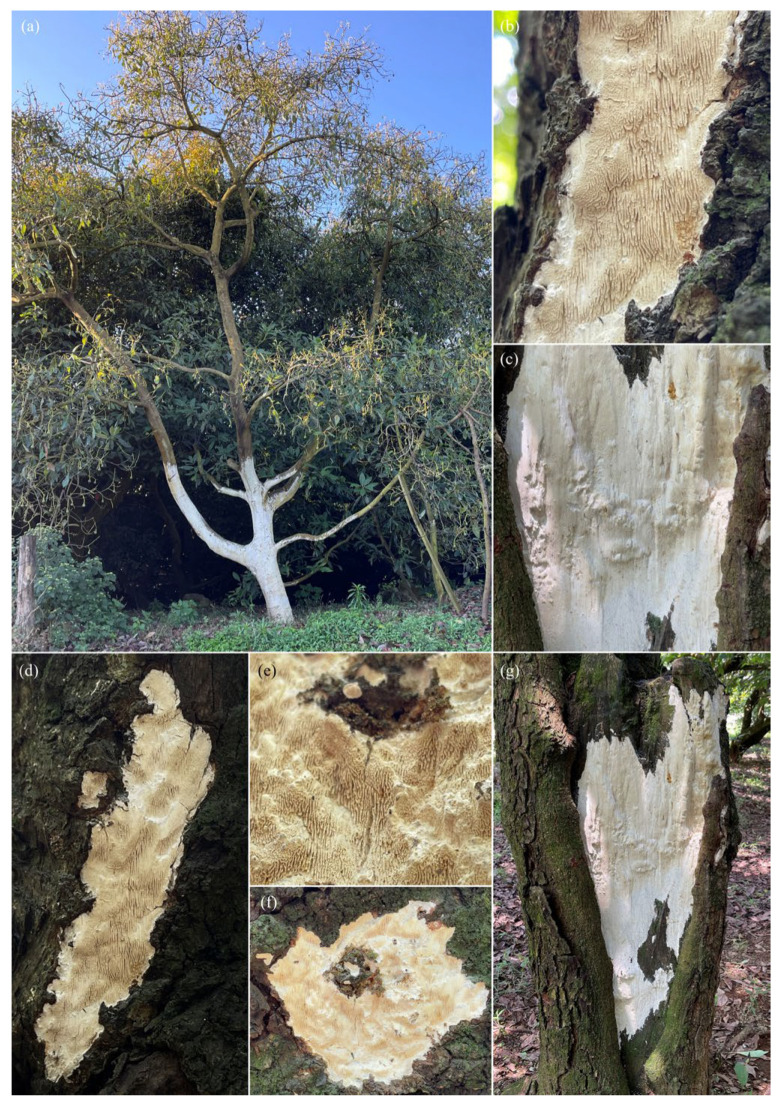
Morphology of *Grammothele* sp.—resupinate basidiomata associated with wood of *Persea americana* var. *Hass*: (**a**) general view of the tree in the field; (**b**) detail of the hymenophore showing a white–cream poroid–irpicoid surface; (**c**) whitening of the exposed xylem beneath the sporoma; (**d**–**f**) resupinate basidiomata developed on the trunk, firmly attached to the substrate; (**e**) detail of the hymenial surface with an irregular pattern characteristic of corticioid fungi; (**g**) extensive colonization area on the trunk with visible degradation of woody tissue.

**Figure 2:**
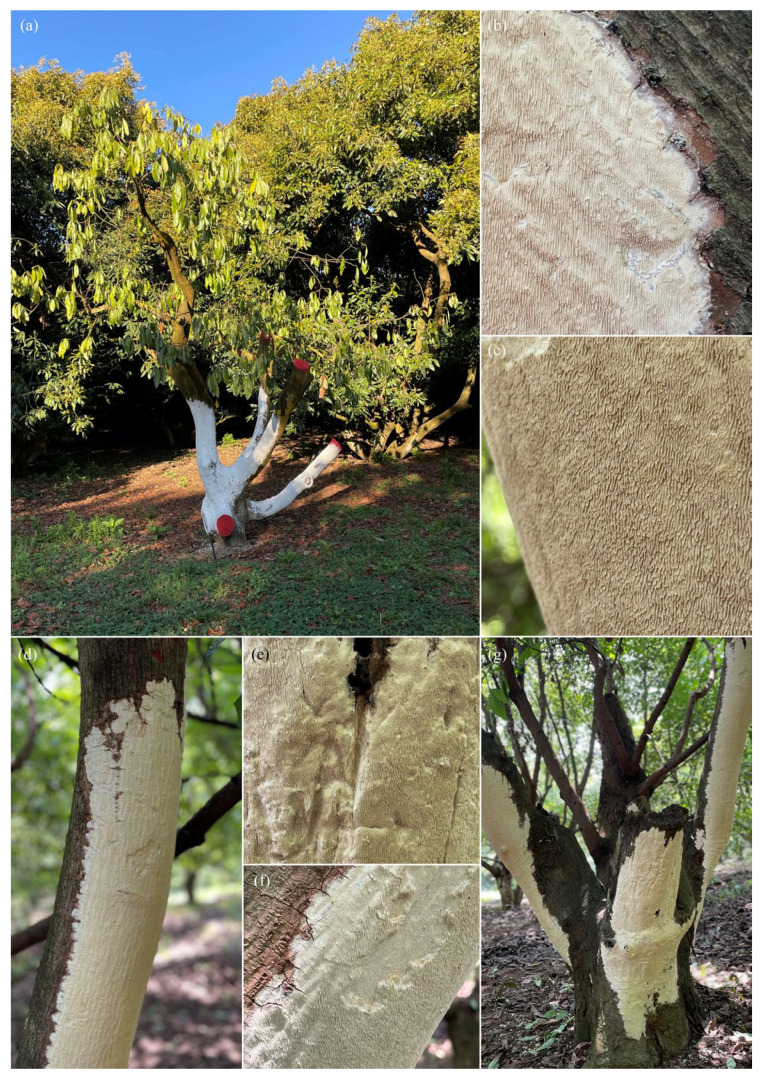
Resupinate basidiomata of *Grammothele* sp. associated with *Persea americana* var. *Hass*: (**a**) general view of the tree showing extensive colonization areas on the trunk; (**b**,**c**) details of the hymenophore with an odontioid surface bearing short, densely arranged dentiform projections; (**d**) white–cream resupinate basidioma firmly attached to the substrate; (**e**,**f**) details of the hymenium displaying a rough and uniform pattern; (**g**) broad colonization area with xylem whitening and visible degradation of woody tissue.

**Figure 3:**
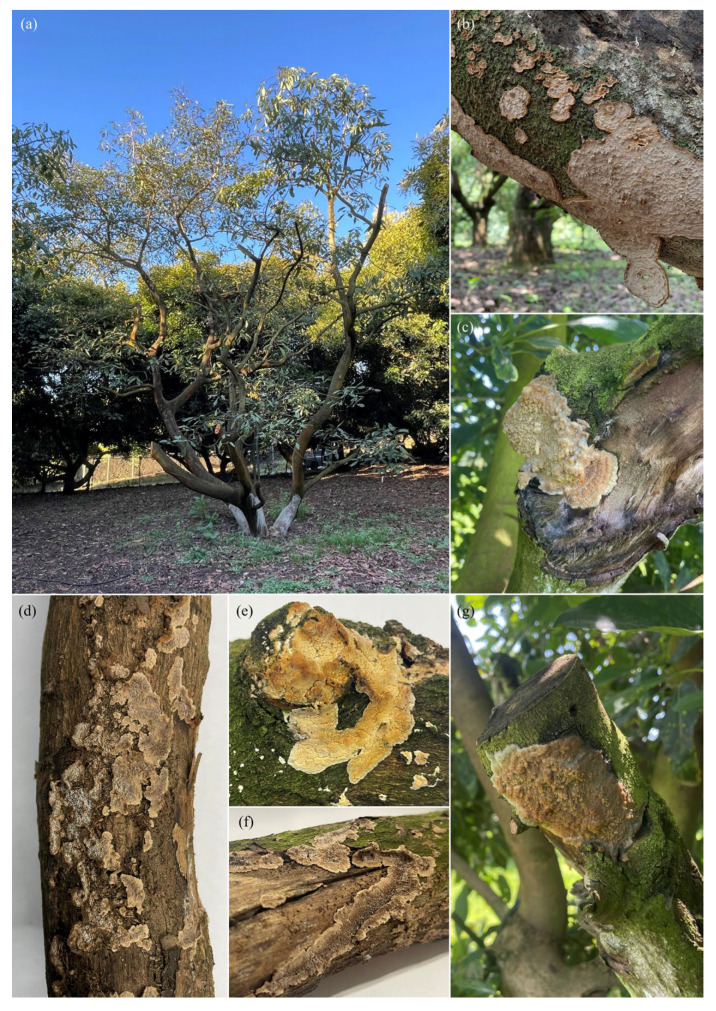
Resupinate basidiomata of *Dentocorticium portoricense* developed on trunks and branches of *Persea americana* var. *Hass*: (**a**) general view of the host tree; (**b**,**c**) basidiomata with irregular growth on branches, cream to yellowish in color; (**d**) discontinuous development of the basidioma on the trunk; (**e**,**f**) details of the odontioid hymenophore with less uniform dentiform projections; (**g**) sporoma associated with pruning wounds, firmly attached to the woody substrate.

In trees showing a higher degree of damage, branch dieback, reduced vegetative vigor, and foliar chlorosis were recorded. In addition, resupinate basidiomata firmly attached to the surface of woody tissues were detected on affected trunks and branches (Figure 1, Figure 2 and Figure 3).

### 3.2. Morphological Characterization of Basidiomata

Basidiomata collected in the field exhibited macromorphological characteristics consistent with corticioid fungi. Basidiomata were resupinate or effused, firmly adherent to the woody substrate, corky in texture when fresh and brittle when dry, with white to pale yellowish margins and smooth to slightly rough surfaces, ranging in color from white to light brown (Figure 1, Figure 2 and Figure 3).

#### 3.2.1. Basidiomata of *Grammothele* sp.

Basidiomata attributed to *Grammothele* sp. corresponded to thin, resupinate basidiomata with a membranous to coriaceous consistency, forming irregular crusts on trunks and branches. The hymenophore exhibited a poroid to irpicoid surface, with shallow pores arranged in angular to sinuous patterns. Hymenial coloration ranged from white to creamy white, without dark brown tones. In longitudinal section, a thin subiculum and short tubes were observed, with the hymenium mainly restricted to the base of the tubes (Figure 1 and Figure 2).

#### 3.2.2. Basidiomata of *Dentocorticium portoricense*

Basidiomata of *Dentocorticium portoricense* developed as extensive resupinate crusts on trunks and branches, mainly in areas exhibiting cortical damage. They displayed a white to creamy-white coloration and a well-defined odontioid hymenophore, with short, densely arranged tooth-like projections oriented perpendicular to the substrate. In adjacent tissues, xylem whitening and a loss of wood consistency were observed, with no evidence of brown rot symptoms (Figure 3).

### 3.3. Recovery of Fungal Isolates

From surveys conducted in 15 commercial orchards within the avocado-producing belt of Michoacán State, a total of 28 pure fungal isolates were obtained from woody tissues and associated basidiomata collected from trees exhibiting white rot symptoms. All isolates showed active mycelial growth on PDA medium, with noticeable variation in growth rate, texture, and mycelial coloration, allowing preliminary differentiation into distinct morphotypes.

**Figure 4:**
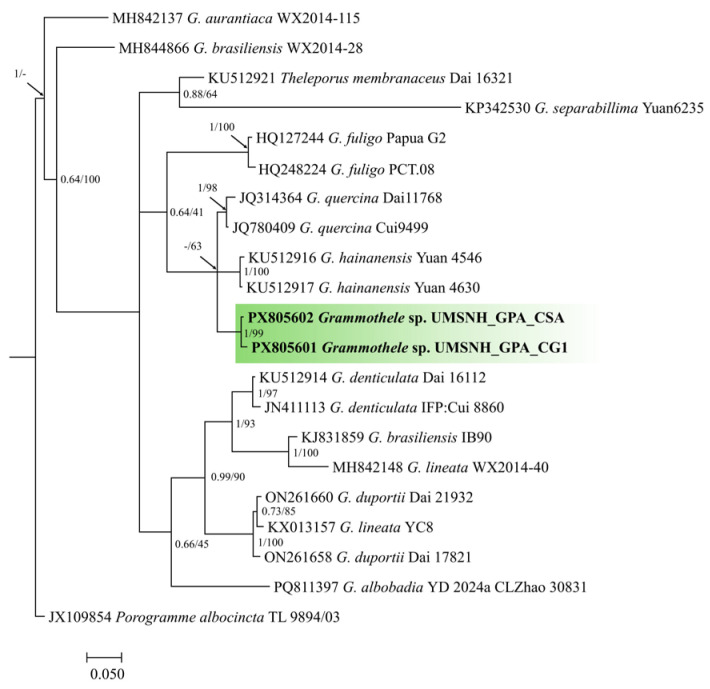
Phylogenetic tree of the genus *Grammothele* based on ITS region sequences obtained by Bayesian inference. Node support values correspond to Bayesian posterior probability and maximum likelihood bootstrap. The isolates analyzed in this study form an independent clade, clearly differentiated from the outgroup *Porogramme albocincta*, with maximum support (PP = 1.0; BS = 99). The scale bar indicates the expected number of substitutions per site. The clade is marked in green to highlight it (differentiate it from the others).

**Figure 5:**
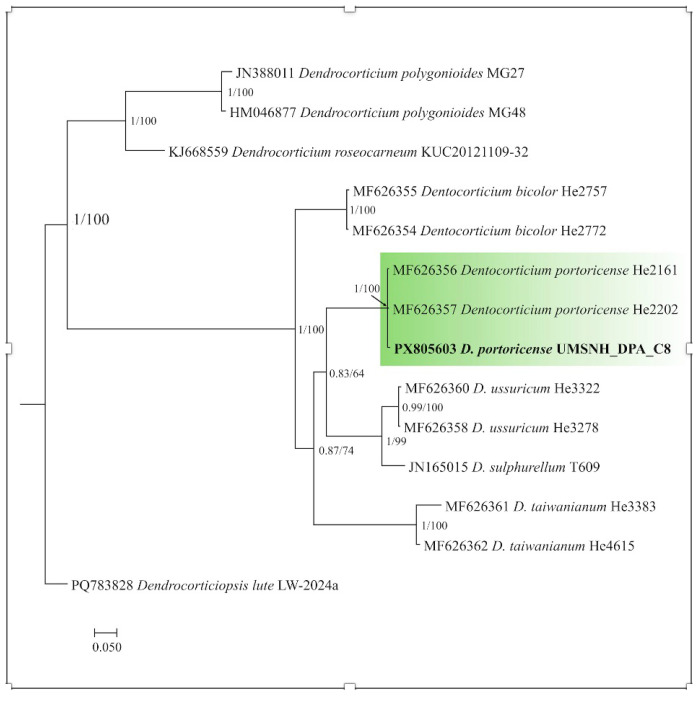
Phylogenetic tree of the genus *Dentocorticium* based on ITS sequences obtained by Bayesian inference. Node support values correspond to Bayesian posterior probability and maximum likelihood bootstrap. The isolate analyzed in this study (C8) is indicated in bold and clusters within the *Dentocorticium portoricense* clade with maximum support (PP = 1.0; BS = 100). The tree was rooted using *Dendrocorticiopsis lutea*, *Dendrocorticium polygonioides*, and *Dendrocorticium roseocarneum* as outgroups. The scale bar indicates the expected number of substitutions per site. The clade is marked in green to highlight it (differentiate it from the others), it contains three active sequences which are the sequences with which it was analyzed (two) and one in bold which belongs to our strain.

**Figure 6:**
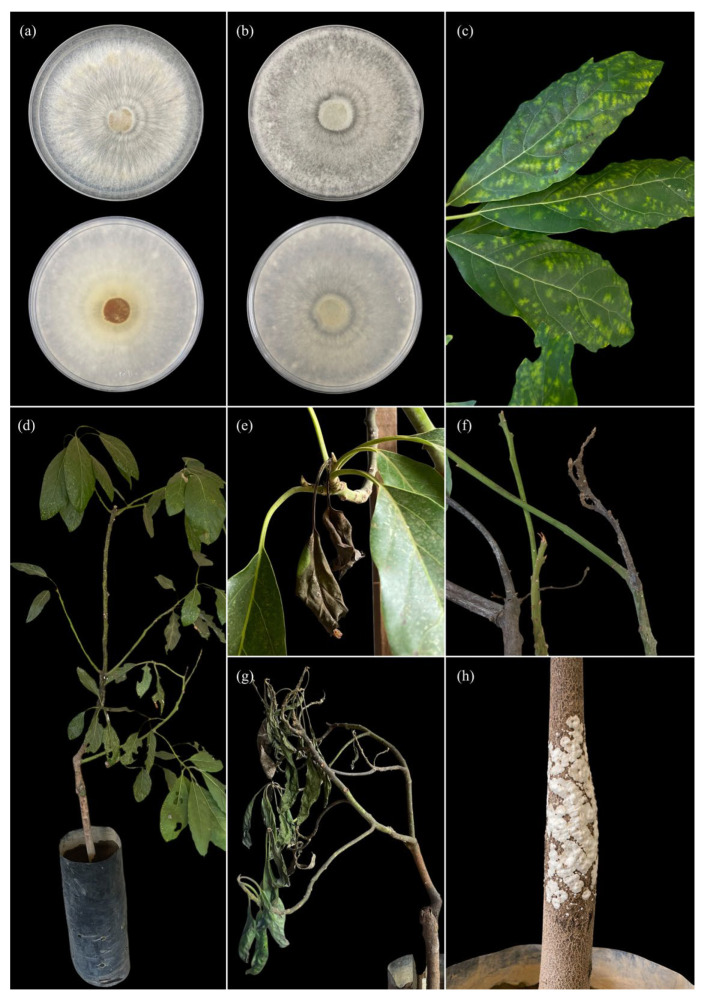
Pathogenicity of *Grammothele* sp.—visual evidence of pathogenicity and associated signs on *Persea americana* var. *Hass*: (**a**) colony growth on Petri dishes (upper/obverse and lower/reverse views), with white to creamy-white mycelium and radial zonation; (**b**) colony growth of a second isolate on Petri dishes (obverse and reverse), showing grayish-white mycelium, faint concentric zonation, and a denser center; (**c**) inoculated leaves exhibiting chlorotic mottling (irregular yellowish spots/patches distributed over the lamina); (**d**) whole seedling showing general decline (reduced vigor) with foliage still present; (**e**) detail of shoot/twig with wilting and foliar necrosis (collapsed, brown, and dry leaves) hanging downward; (**f**) twigs with shoot dieback and progressive desiccation of the apical end; (**g**) seedling with severe wilting and collapse of the aerial part (flaccid and dehydrated foliage); (**h**) seedling stem with external signs: whitish, crustose superficial growth adhered to the bark (visible surface colonization).

**Figure 7:**
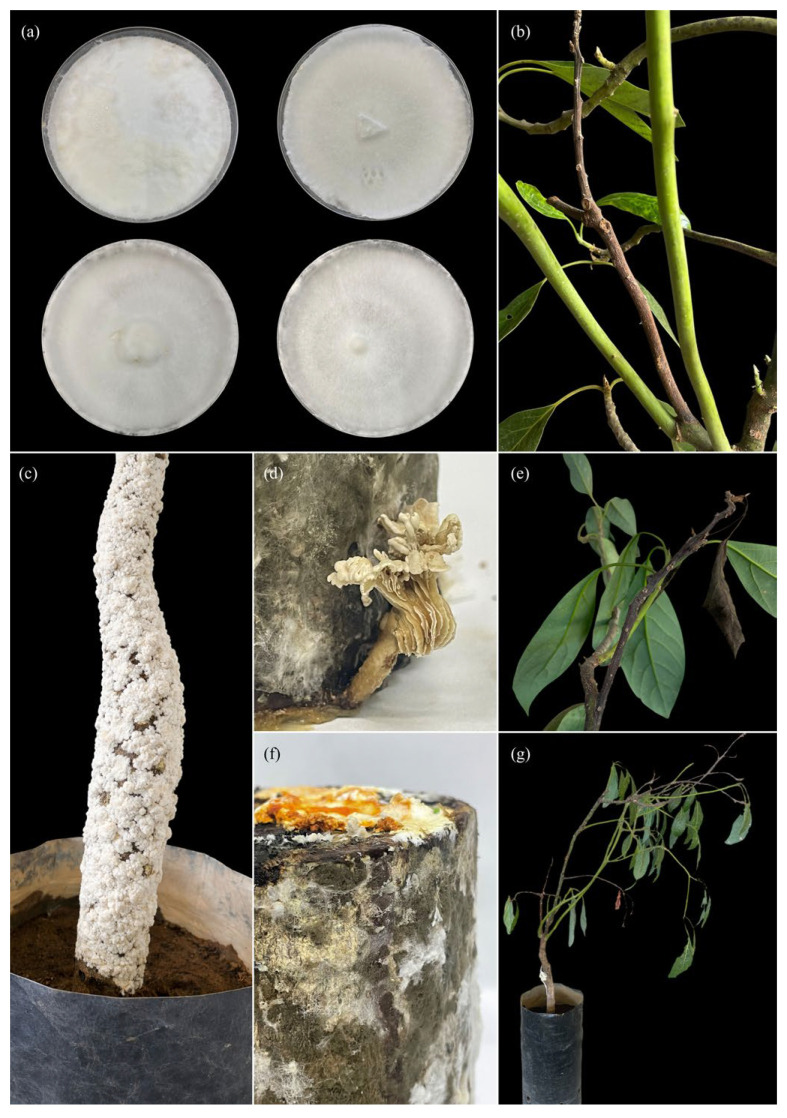
Symptoms and signs observed in pathogenicity tests with *Grammothele* sp. on *Persea americana* var. *Hass* seedlings and woody substrates: (**a**) colonies on culture medium (four Petri dishes) with white mycelium and homogeneous growth, showing variation in density at the central area among replicates; (**b**) twigs and stems with localized necrosis and segmental shoot dieback (brown, dry tissue in portions of the shoot); (**c**) seedling stem with abundant external colonization, visible as a white, granular coating covering much of the cortical surface (sign); (**d**) woody substrate with emerging fungal structures (three-dimensional swellings/lobes) on the incubated material (sign); (**e**) branch with advanced necrosis and desiccation (dark/brown segments) with affected leaves; (**f**) incubated woody substrate showing visible mycelial growth and yellowish–orange exudate/pigmentation on the upper surface (sign and substrate change); (**g**) seedling with general decline, wilting, and marked reduction in vigor (drooping, dehydrated foliage).

**Figure 8:**
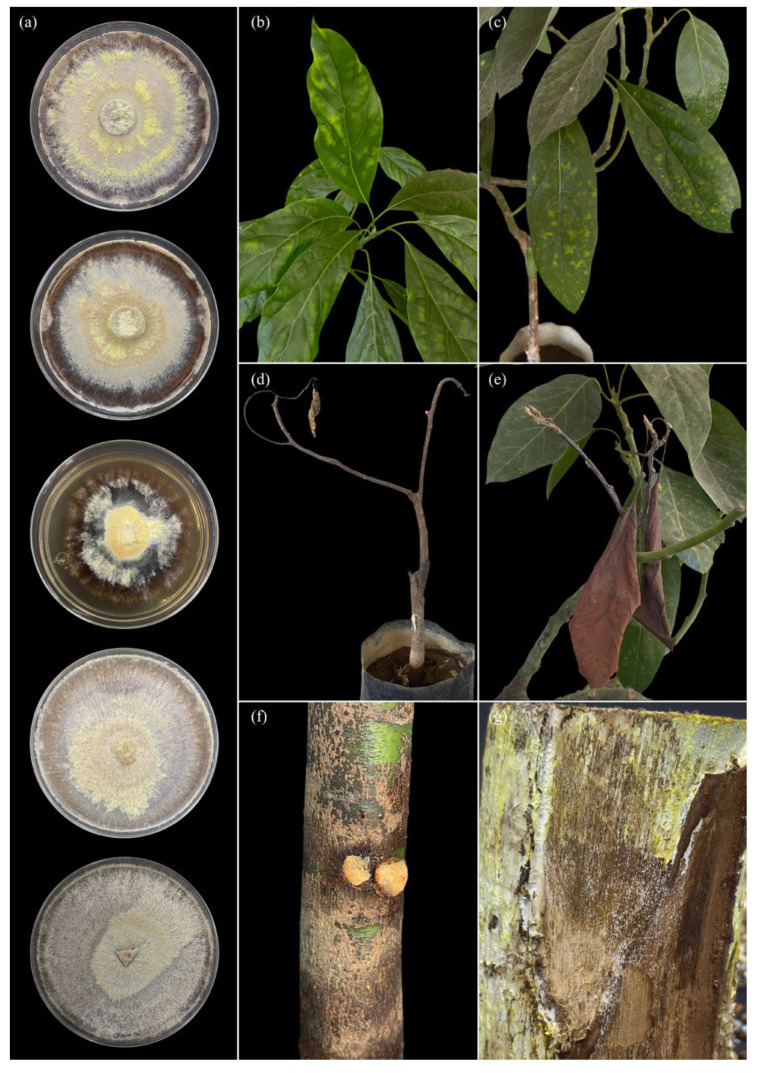
Symptoms and signs observed in pathogenicity tests of *Dentocorticium portoricense* on *Persea americana* var. *Hass* and its growth in culture: (**a**) colonies on culture medium (five Petri dishes) with marked zonation, showing concentric areas of creamy-white mycelium and sectors with yellowish to brown pigmentation; variation among plates is evident; (**b**) seedling/twig without evident symptoms (green, turgid leaves), serving as a visual reference of the unaffected state or early stage; (**c**) leaves with chlorotic mottling (dispersed yellowish spots) on the lamina; (**d**) seedling with severe shoot dieback and defoliation/collapse of the aerial part (dry branches with marked foliage loss); (**e**) detail of foliar necrosis (brown, dry leaves) associated with declining twigs; (**f**) stem with lesions/inoculation areas visible as two circular light-colored points (marked sites on the bark, compatible with inoculation points); (**g**) exposed woody tissue with discoloration and visible surface alteration (brown areas and pale/yellowish zones), with superficial material compatible with fungal colonization on the exposed area.

**Figure 9:**
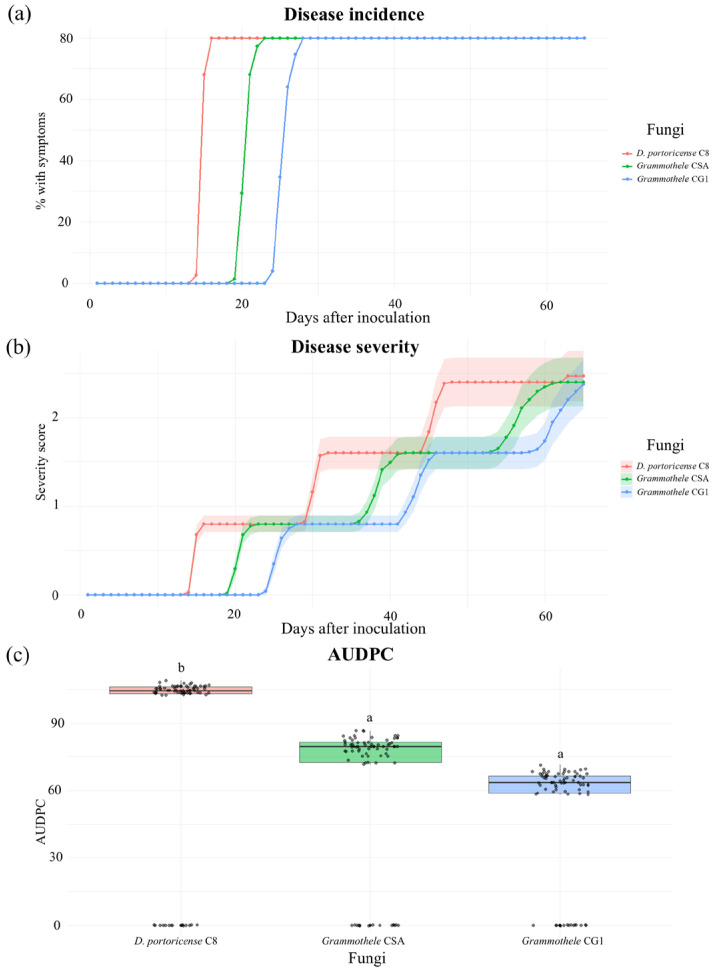
Pathogenicity assessment of corticioid fungal isolates inoculated on avocado seedlings (*Persea americana* var. *Hass*): (**a**) disease incidence, expressed as the percentage of inoculated plants that developed visible symptoms; (**b**) disease severity, estimated according to the extent of tissue damage observed on each plant; (**c**) aggressiveness of the isolates, quantified as the area under the disease progress curve (AUDPC).

Based on the morphological diversity observed both in the field and in culture, ten representative isolates were selected for the first experimental block. These isolates originated from different municipalities within the region (Ziracuaretiro, Salvador Escalante, Puruarán, Parácuaro, Tancítaro, and Uruapan), in order to capture the geographical and morphological variability of the recovered fungi. Isolates associated with the corticioid morphological group were selected based on their delimitation using the BLAST tool (BLAST+ 2.17.0) of the NCBI, retaining three isolates (CSA, CG1, and C8) for identification through phylogenetic reconstruction (Figure 4 and Figure 5) and for subsequent pathogenicity (Figure 6, Figure 7 and Figure 8) and aggressiveness assays (Figure 9).

### 3.4. Morphological Characterization of Isolates in Culture

On PDA medium, the selected isolates (CSA, CG1, and C8) exhibited slow to moderate growth, with predominantly white to creamy-white mycelium in isolates CSA and CG1 (Figure 6 and Figure 7), and in some cases light brown tones in isolate C8 (Figure 6). Colonies showed a cottony, velvety, or slightly flattened texture and were firmly adherent to the medium. Colony margins were regular to slightly irregular, with no evident production of diffusible pigments or exudates. Micromorphological analysis revealed hyaline, septate hyphae with clearly visible clamp connections in most isolates. Hyphae exhibited thin to moderately thickened walls, frequent branching, and variable diameters. No anamorphic structures or conidial production were observed under in vitro conditions. In some isolates, generative and skeletal hyphae were identified, suggesting a dimitic hyphal system, characteristic of white-rot-associated basidiomycetes.

In *Grammothele* isolates, colonies developed on culture media with uniform radial growth, white to creamy-white cottony mycelium (Figure 6a), and concentric growth patterns (Figure 7a,b). No macroscopic reproductive structures were observed during the evaluation period.

In the case of *Dentocorticium portoricense*, colonies grown in culture exhibited dense white mycelium, with homogeneous growth and continuous radial expansion, uniformly covering the surface of the culture medium (Figure 8a).

### 3.5. Identification Through Phylogenetic Reconstruction

Molecular analysis based on the ITS region enabled the identification of three corticioid fungal isolates: two belonging to the genus *Grammothele* (CG1 and CSA) and one to the genus *Dentocorticium* (C8). In the phylogenetic analysis of *Grammothele*, both isolates clustered within a well-supported clade (PP/BS = 1.0/99), clearly differentiated from the outgroup *Porogramme albocincta* (Figure 4).

In the case of *Dentocorticium*, isolate C8 was consistently grouped within the clade of *Dentocorticium portoricense*, showing maximum support in both Bayesian inference and maximum likelihood analyses (PP/BS = 1.0/100), and was clearly separated from other lineages of the genus included in the analysis (Figure 5).

### 3.6. Pathogenicity Test Results

Inoculated avocado seedlings developed the first symptoms at 82 days post inoculation, initially manifested as foliar chlorosis and progressive apical necrosis. Subsequently, from 125 days onward, creamy-white mycelial growth with a corky appearance was observed on the substrate, collar region, and cotyledons of the inoculated plants.

Pathogenicity assays conducted on avocado seedlings inoculated with corticioid fungi revealed the progressive development of foliar and stem symptoms, alterations of woody tissues, and the presence of signs associated with mycelial growth, both under in vitro conditions and in plants inoculated under controlled conditions (Figure 6, Figure 7 and Figure 8).

In seedlings inoculated with *Grammothele*, initial symptoms appeared as irregular chlorotic mottling distributed across the leaf lamina in both young and mature leaves (Figure 6c,d). As the evaluation period progressed, these symptoms advanced to partial foliar wilting (Figure 6e,g), characterized by loss of turgor and leaf curvature, followed by foliar necrosis, mainly affecting leaf apices and margins (Figure 6f,g and Figure 7d). At advanced stages, shoot dieback was observed, with progressive desiccation of lateral branches and a visible reduction in seedling vegetative vigor (Figure 6f and Figure 7e).

At the stem level, plants inoculated with *Grammothele* exhibited localized signs characterized by the presence of visible resupinate basidiomata on affected stem areas (Figure 6h). Longitudinal stem sections revealed alterations in woody tissues, including loss of xylem consistency and changes in internal coloration, without evidence of generalized darkening or cubical cracking of the wood.

In plants inoculated with isolate C8, more severe foliar symptoms were observed, beginning with pronounced chlorotic mottling and generalized chlorosis of the leaf lamina (Figure 6b,c). Subsequently, advanced wilting was recorded, characterized by collapse of foliar tissues, followed by branch necrosis, foliage desiccation, and evident shoot dieback (Figure 6d,e). In addition, clear signs of the pathogen were detected on stems inoculated with *D. portoricense*, including abundant external colonization characterized by firmly adherent mycelial growth on the cortical surface, particularly around the inoculation point (Figure 6f). Longitudinal stem sections revealed whitening of woody tissues, structural alteration of the xylem, and a marked loss of tissue consistency, evidencing progressive wood degradation associated with fungal colonization (Figure 6g).

Taken together, Figure 6, Figure 7 and Figure 8 visually document the sequence of symptom onset and progression in leaves, stems, and woody tissues, as well as the presence of mycelial signs, thereby supporting the quantitative results of incidence, severity, and aggressiveness obtained in the pathogenicity evaluations.

### 3.7. Incidence, Severity, and Aggressiveness

The isolate *Dentocorticium portoricense* (C8) exhibited an earlier and more advanced temporal incidence compared with the *Grammothele* isolates (CSA and CG1), which began to show symptoms from 20 days after inoculation (dai) (Figure 9a).

Regarding disease severity, *D. portoricense* C8 recorded the highest level of tissue damage (84.0 ± 3.96), followed by *Grammothele* CSA (63.8 ± 3.96) and *Grammothele* CG1 (51.8 ± 3.96) (Figure 9b).

Statistically significant differences were detected among the evaluated isolates in terms of aggressiveness (*p* < 0.05). Aggressiveness analysis, determined through the calculation of the area under the disease progress curve (AUDPC), showed a similar pattern consistent with incidence and severity, with significantly higher aggressiveness values for *D. portoricense* C8 compared with the *Grammothele* isolates (CSA and CG1) (Figure 9c).

## 4. Discussion

The evidence obtained in this study demonstrates that corticioid fungi associated with white rot of woody tissues act as active and differential pathogenic role in the progressive deterioration of stems and branches of *Persea americana* var. *Hass* within the avocado-producing belt of Michoacán, Mexico.

The consistent presence of structural signs and symptoms observed under field conditions, together with pathogenicity, confirmation under controlled conditions and phylogenetic support of the isolates, allows us to establish that these basidiomycetes should not be regarded solely as secondary saprophytes, but rather as functional phytopathogenic agents within intensive agricultural systems, particularly in avocado production systems.

In the evaluated orchards, the observed symptoms included structural weakening of branches, mechanical fractures, foliar chlorosis, shoot dieback, and exposure of whitish wood with a fibrous to spongy texture, which unequivocally correspond to white rot processes. This type of deterioration is characterized by the preferential degradation of lignin and the subsequent disorganization of xylem structural components, leading to a significant loss of mechanical resistance of the tree [7,11,20]. The absence of generalized darkening or cubical cracking in affected wood allowed brown rot processes to be ruled out and reinforced the association with ligninolytic basidiomycetes [21].

A relevant aspect observed under field conditions was the discrepancy between the severity of internal damage and the external expression of symptoms, a phenomenon widely documented in chronic woody tissue diseases. In such pathosystems, internal colonization and degradation may progress over prolonged periods before visible symptoms become evident in the aerial parts of the tree, which explains the delayed appearance of dieback, defoliation, or structural collapse in apparently functional trees [17]. In this context, the detection of resupinate basidiomata firmly attached to stems and branches acquires high diagnostic value, as it represents one of the few visible indicators of advanced colonization of woody tissues by corticioid fungi.

Morphological characterization of basidiomata and cultures allowed the recognition of features consistent with the genera *Grammothele* and *Dentocorticium*, including resupinate basidiomata firmly attached to the substrate, pale hymenial surfaces, and distinctive hymenial patterns. Nevertheless, the high morphological plasticity of corticioid fungi, together with the scarcity of highly diagnostic macroscopic characters, limits species-level identification based solely on morphology [22,23]. In this regard, the results of the present study reinforce the need to integrate molecular and phylogenetic approaches to achieve reliable taxonomic delimitation in phytopathological studies involving basidiomycetes with corticioid morphology.

Pathogenicity tests confirmed that the evaluated isolates are capable of inducing symptoms consistent with those observed under field conditions, strengthening the causal relationship between the isolated fungi and the woody tissue decline syndrome in avocado. However, quantitative analyses revealed significant differences in incidence, severity, and aggressiveness among the evaluated taxa. In particular, the isolate identified as *Dentocorticium portoricense* (C8) exhibited the highest severity and AUDPC values, indicating greater aggressiveness, understood as the combination of damage magnitude and the rate of disease progression over time [15]. In contrast, *Grammothele* sp. isolates (CSA and CG1) showed a slower and more moderate development of disease.

These differences in aggressiveness may be related to functional variation in the ligninolytic capacity of the isolates. White-rot basidiomycetes possess highly specialized enzymatic complexes, mainly composed of laccases, manganese-dependent peroxidases, and lignin peroxidases, which enable the efficient degradation of lignin and other structural polymers of the plant cell wall [24,25,26]. Although enzymatic activity was not directly evaluated in the present study, the observed severity and AUDPC patterns are consistent with a higher ligninolytic efficiency of isolate C8 compared with *Grammothele* isolates.

From a molecular perspective, ITS-based identification allowed robust phylogenetic assignment of isolate C8 as *Dentocorticium portoricense*, with maximum support in both Bayesian inference and maximum likelihood analyses. In the case of *Grammothele*, isolates CSA and CG1 clustered consistently within the genus but lacked unequivocal species-level resolution. This limitation is consistent with reports for several Basidiomycota groups, in which the ITS marker exhibits reduced interspecific variability, hindering fine taxonomic delimitation [27,28]. Consequently, the present results suggest that future studies aimed at species-level resolution in *Grammothele* should incorporate additional molecular markers or multilocus approaches, as well as more detailed morphofunctional characterization.

From a phytosanitary perspective, the identification of corticioid fungi with differing pathogenic aggressiveness has relevant implications for avocado orchard management. The chronic and slow-progressing nature of white rot hampers early detection, favoring scenarios in which internal damage advances unnoticed until severe structural failure or significant productivity losses occur. In this context, the recognition of *D. portoricense* as a highly aggressive agent and *Grammothele* spp. as more slowly progressing pathogens provides a scientific basis for the design of monitoring strategies, early diagnostic tools, and preventive management approaches targeting woody tissue diseases in avocado.

Overall, the results obtained support the notion that corticioid fungi, traditionally regarded as strictly saprophytic organisms, may modify their ecological behavior and act as facultative pathogens in intensive agricultural systems. The expansion of avocado cultivation in regions with high diversity of lignicolous fungi, combined with management practices and climatic factors that generate wounds in woody tissues, may favor this functional shift. Corticioid fungi as emerging or previously underestimated pathogens in avocado phytosanitary systems.

## 5. Conclusions

This study provides strong evidence that corticioid fungi associated with white rot of avocado woody tissues act as active pathogens rather than secondary saprophytes. Field observations, morphological and molecular analyses, and pathogenicity tests consistently confirmed their role in stem and branch decline. Among the evaluated taxa, *Dentocorticium portoricense* exhibited significantly higher incidence, severity, and aggressiveness compared to *Grammothele* spp., underscoring its relevance as a major phytopathogenic agent in avocado orchards. These findings highlight the importance of incorporating corticioid fungi into diagnostic protocols, monitoring systems, and management strategies for woody tissue diseases. Recognizing their pathogenic potential will improve early detection and strengthen phytosanitary measures, ultimately reducing structural damage and productivity losses in avocado production systems.

## Figures and Tables

**Table 1:** Collection sites of corticioid spores associated with white rot symptoms in avocado orchards across different municipalities within the avocado-producing belt of Michoacán, Mexico.

Municipality	Altitude (msnm)	Precipitation (mL/m^2^)	Geographic Coordinates	Strain
Ziracuaretiro	1345	1100	19.3500 N, 102.5038 W	CSA *
Salvador Escalante	2220	780	19.4083 N, 101.6397 W	CF32
				CF33
				CF35
Puruaran	1097	850	19.0925 N, 101.5213 W	C8 *
Paracuaro	602	800	19.1463 N, 102.2186 W	CB2
	608	800	19.1544 N, 102.2180 W	C57
Tancítaro	1403	800	19.3500 N, 102.3627 W	CG1 *
	1884	1200	19.4260 N, 102.4473 W	C15
Uruapan	1770	1500	19.3717 N, 101.9993 W	C20

Strains marked in bold and with an asterisk (*) indicate cortisone isolates.

## Data Availability

The original contributions presented in this study are included in the article. Further inquiries can be directed to the corresponding author.
